# Reproductive System Symbiotic Bacteria Are Conserved between Two Distinct Populations of *Euprymna scolopes* from Oahu, Hawaii

**DOI:** 10.1128/mSphere.00531-17

**Published:** 2018-03-28

**Authors:** Allison H. Kerwin, Spencer V. Nyholm

**Affiliations:** a Department of Molecular and Cell Biology, University of Connecticut, Storrs, Connecticut, USA; University of Wisconsin—Madison

**Keywords:** 16S rRNA, *Euprymna*, community analysis, host-microbe interactions, symbiosis

## Abstract

Female Hawaiian bobtail squid, Euprymna scolopes, harbor a symbiotic bacterial community in a reproductive organ, the accessory nidamental gland (ANG). This community is known to be stable over several generations of wild-caught bobtail squid but has, to date, been examined for only one population in Maunalua Bay, Oahu, HI. This study assessed the ANG and corresponding egg jelly coat (JC) bacterial communities for another genetically isolated host population from Kaneohe Bay, Oahu, HI, using 16S amplicon sequencing. The bacterial communities from the ANGs and JCs of the two populations were found to be similar in richness, evenness, phylogenetic diversity, and overall community composition. However, the Kaneohe Bay samples formed their own subset within the Maunalua Bay ANG/JC community. An *Alteromonadaceae* genus, BD2-13, was significantly higher in relative abundance in the Kaneohe Bay population, and several *Alphaproteobacteria* taxa also shifted in relative abundance between the two groups. This variation could be due to local adaptation to differing environmental challenges, to localized variability, or to functional redundancy among the ANG taxa. The overall stability of the community between the populations further supports a crucial functional role that has been hypothesized for this symbiosis.

**IMPORTANCE** In this study, we examined the reproductive ANG symbiosis found in two genetically isolated populations of the Hawaiian bobtail squid, Euprymna scolopes. The stability of the community reported here provides support for the hypothesis that this symbiosis is under strong selective pressure, while the observed differences suggest that some level of local adaptation may have occurred. These two host populations are frequently used interchangeably as source populations for research. Euprymna scolopes is an important model organism and offers the opportunity to examine the interplay between a binary and a consortial symbiosis in a single model host. Understanding the inherent natural variability of this association will aid in our understanding of the conservation, function, transmission, and development of the ANG symbiosis.

## Abstract (precis)

In this study, we examined the reproductive ANG symbiosis found in two genetically isolated populations of the Hawaiian bobtail squid, Euprymna scolopes. The stability of the community reported here provides support for the hypothesis that this symbiosis is under strong selective pressure, while the observed differences suggest that some level of local adaptation may have occurred. These two host populations are frequently used interchangeably as source populations for research. Euprymna scolopes is an important model organism and offers the opportunity to examine the interplay between a binary and a consortial symbiosis in a single model host. Understanding the inherent natural variability of this association will aid in our understanding of the conservation, function, transmission, and development of the ANG symbiosis.

## INTRODUCTION

**FIG 1:**
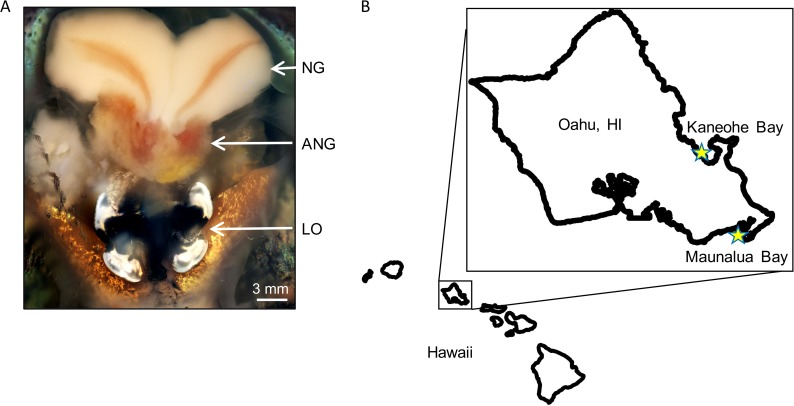
(A) Ventral dissection of adult female squid showing the nidamental glands (NG), accessory nidamental gland (ANG), and light organ (LO). (B) Map of Oahu, HI, showing locations of two squid populations, Kaneohe Bay (21°25′44.0″N, 157°47′32.4″W) and Maunalua Bay (21°16′51.42″N, 157°43′33.07″W).

The bobtail squid, Euprymna scolopes, is endemic to the Hawaiian archipelago and relies on the well-studied light organ symbiont Vibrio fischeri to avoid predation (1). In addition to this symbiosis, adult females also harbor a complex bacterial consortium in their reproductive system (2, 3). The accessory nidamental gland (ANG) is conserved throughout many species of squid, cuttlefish, and bobtail squid (4) and was first described over a century ago (5). This gland consists of a number of epithelium-lined tubules, each of which contains its own dominant bacterial taxon (Fig. 1A) (2). The ANG bacteria are deposited into the jelly coat (JC) of eggs (3), where they are hypothesized to be involved in the defense of developing embryos from microbial fouling (2, 6, 7). Bacterial isolates from the ANG have also been demonstrated to inhibit certain marine bacteria via the production of secondary metabolites (7, 8).

Research into these bobtail squid and their symbionts has primarily focused on two populations of E. scolopes from the island of Oahu, HI (Fig. 1B). Maunalua Bay (MB), located on the southern coast of Oahu, is a shallow sand flat reaching approximately 600 m from shore to the reef crest. Kaneohe Bay (KB) is located to the north of MB on the eastern coast of Oahu and is the only true barrier reef in the Hawaiian archipelago (9). While bobtail squid are found in several discrete spots throughout KB (10), they are generally collected from a smaller sand flat reaching approximately 120 m from shore to a deeper channel.

These two bobtail squid host populations are located on the same island (Fig. 1B) but exhibit low levels of gene flow and several morphological differences (11). MB females, eggs, and juveniles are significantly larger than those from KB (11). These MB and KB hosts are also known to harbor different strains of V. fischeri in their light organs (12), although no evidence for geographic specificity of the strains from these two bobtail squid populations has been found (13), and bacterial lineages from MB and KB hosts show extensive mixing (14). The low levels of gene flow between host populations, along with previously described light organ symbiont strain differences between these two sites, make these populations a good source for examining potential variation in the ANG symbiotic communities.

We hypothesized that the ANGs from these two host populations would contain similar bacterial communities with minor variations, similar to what is seen for the light organ symbiosis. For this study, we collected squid from KB and compared the ANG and egg jelly coat (JC) communities to previously published samples from MB animals (3). We also compared the JC communities to the ANGs from females associated with those eggs, to confirm whether symbionts from mother (ANG) and corresponding egg (JC) communities clustered together, as had previously been demonstrated for the MB population (3).

## RESULTS

**FIG 2:**
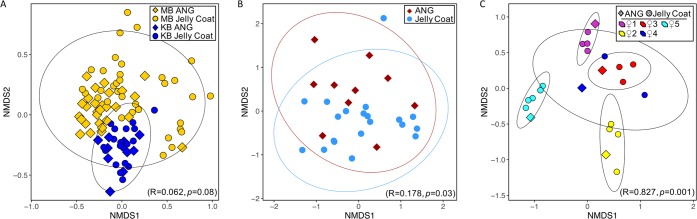
Bray-Curtis beta diversity analysis of Kaneohe Bay (KB) ANG and JC bacterial communities. (A) The overall community composition of KB and Maunalua Bay (MB) ANGs and JCs overlapped, but the KB samples had lower dispersion and clustered apart from the MB samples. (B) ANG and JC bacterial community compositions were not distinct in Kaneohe Bay. (C) KB ANG and JC samples clustered by associated female. Ellipses represent 95% confidence intervals. Results of ANOSIM are presented in parentheses in each plot.

To examine the natural variability of the E. scolopes ANG bacterial community, the V4 region of the 16S rRNA gene from ANG and JC bacterial extracts from Kaneohe Bay was sequenced and compared to previously published samples from Maunalua Bay (3). The bacterial communities from Kaneohe Bay ANGs and JCs clustered together, overlapping with the Maunalua Bay ANGs and JCs but with lower dispersion in a distinct cluster in a Bray-Curtis beta diversity analysis of community composition variation (Fig. 2A). An analysis of similarity (ANOSIM) indicated no dissimilarity between Kaneohe Bay and Maunalua Bay bobtail squid ANG and JC community composition (*R* = 0.06, *P* = 0.08), while a permutational multivariate analysis of variance (PERMANOVA) did find low levels of dissimilarity between the two populations (*F* = 6.07, *P* = 0.001). PERMANOVA is known to be more sensitive to variation in dispersion, and the significance of this test is thus likely due to the lower dispersion of the Kaneohe Bay ANG/JC samples. Kaneohe Bay ANG and JC samples clustered together on a nonmetric multidimensional scaling (NMDS) plot based on the Bray-Curtis metric (Fig. 2B) (ANOSIM: *R* = 0.18, *P* = 0.03; PERMANOVA: *F* = 3.06, *P* = 0.02), in agreement with a previous study, which found a similar pattern for Maunalua Bay ANG and JC samples (3). When compared, the bacterial community composition of Kaneohe Bay JCs reflected that of the ANG of the female that deposited the eggs, with a strong cluster pattern (Fig. 2C) (ANOSIM: *R* = 0.83, *P* = 0.001; PERMANOVA: *F* = 7.37, *P* = 0.001), similar to what was shown previously for the Maunalua Bay population (3).

**FIG 3:**
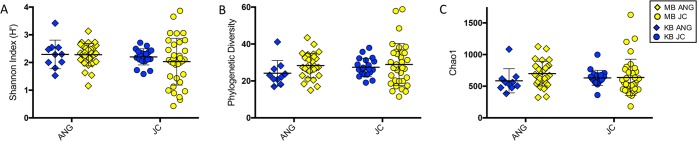
Alpha diversity analysis of Kaneohe Bay ANG and JC bacterial communities. Two-way ANOVA (see Table S1 in the supplemental material) revealed no effect of population or tissue type on bacterial community richness/evenness (A), phylogenetic diversity (B), or richness informed by the number of rare taxa present (C). Thick bars indicate means; thin bars indicate standard deviations.

Alpha diversity was also similar between KB and MB populations, for both ANG and JC bacterial communities (Fig. 3). Three types of alpha diversity were analyzed to give a broad portrait of within-sample diversity. Bacterial richness and evenness (H′), phylogenetic diversity (PD), and richness informed by the number of rare operational taxonomic units (OTUs, Chao1) were all similar between the two populations and two sample types, when analyzed via two-way ANOVA (Fig. 3). The larger spread in alpha diversity of MB JC samples than of other sample types is attributed to including a wider set of JCs from different stages of embryogenesis in the initial study (3), while this study included JCs only from eggs collected during early embryogenesis.

10.1128/mSphere.00531-17.1 TABLE S1  Two-way ANOVA of alpha diversity analysis of Kaneohe Bay ANGs and JCs (Fig. 3) found no effect of population or sample type on bacterial community richness/evenness (A), phylogenetic diversity (B), or richness informed by the number of rare taxa present (C). Thick bars indicate means; thin bars indicate standard deviations. Download TABLE S1, TIF file, 0.2 MB. Copyright © 2018 Kerwin and Nyholm. 2018 Kerwin and Nyholm This content is distributed under the terms of the Creative Commons Attribution 4.0 International license.

**FIG 4:**
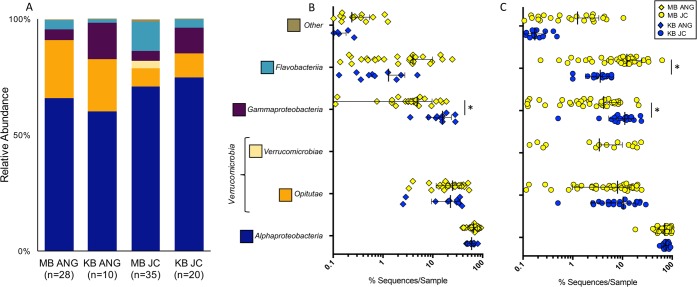
(A) Kaneohe Bay (KB) ANG and JC bacterial communities resembled those observed in Maunalua Bay (MB) at the class level. (B) KB animals had significantly more *Gammaproteobacteria* in their ANGs than MB hosts (*t*_36_ = 5.129, *P* < 0.0001). (C) JCs from KB animals had significantly more *Gammaproteobacteria* than MB JCs (*t*_53_ = 4.73, *P* = 0.0001) and also fewer *Flavobacteriia* (*t*_53_ = 3.138, *P* = 0.01). Taxa are presented at the class level (*Verrucomicrobiae* and *Opitutae* are both classes within the *Verrucomicrobia* phylum); the scatter plot is presented on a log scale to show variation for taxa present at lower average abundances. Thick bars represent means; thin bars represent standard deviations; asterisks represent significant differences between populations (B and C). Error bars that would have extended below 0.1% sequences/sample have been omitted from the graph (B and C). The “other” component included taxa present in more than one sample and at less than 0.3% average abundance.

**FIG 5:**
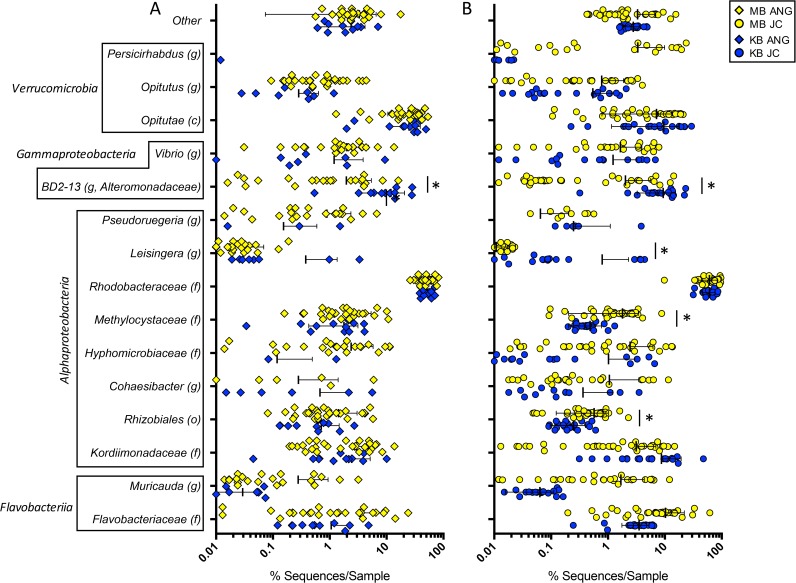
A higher abundance of *Gammaproteobacteria* in the Kaneohe Bay (KB) ANG and JC bacterial communities was due to a shift in BD2-13, a genus from the *Alteromonadaceae* family. BD2-13 (*t*_38_ = 5.22, *P* = 0.0001) was significantly more abundant in KB ANGs (A) and in KB JCs (*t*_53_ = 5.612, *P* = 0.00001) (B). One *Alphaproteobacteria* taxon (*Leisingera*: *t*_53_ = 3.085, *P* = 0.04) was also significantly higher in KB JCs (B), while two others were significantly lower in KB JCs (*Rhizobiales*: *t*_53_ = 3.107, *P* = 0.04; *Methylocystaceae*: *t*_53_ = 3.639, *P* = 0.009). Taxa are presented at the finest level obtained (c, class; o, order; f, family; g, genus); the scatter plot is presented on a log scale to show variation for taxa present at lower average abundances. Thick bars represent means; thin bars represent standard deviations; asterisks represent significant differences between populations. Error bars that would have extended below 0.01% sequences/sample have been omitted from the graph (A and B). The “other” component included taxa present in more than one sample and at less than 0.3% average abundance.

The cluster patterns found via beta diversity analysis between the Kaneohe and Maunalua Bay ANG and JC bacterial communities can be explained by the observed differences in the relative abundances of certain taxa. The KB and MB ANGs were both dominated by *Alphaproteobacteria* (60.3% KB versus 65.9% MB) and *Verrucomicrobia* (22.6% KB versus 25.0% MB) (Fig. 4A). The JCs from both populations had higher levels of *Alphaproteobacteria* (74.8% KB versus 70.9% MB) and lower levels of *Verrucomicrobia* (10.3% KB versus 11.4% MB) than the ANGs (Fig. 4A). However, *Gammaproteobacteria* in KB ANGs accounted for a significantly higher proportion of the community than in MB ANGs (15.7% KB versus 4.9% MB, *t*_34_ = 4.635, *P* = 0.0002) (Fig. 4B). The higher proportion of *Gammaproteobacteria* in KB was due to an *Alteromonadaceae* genus (BD2-13, 11.9% KB versus 2.0% MB, *t*_34_ = 5.023, *P* = 0.0003) (Fig. 5A). A similar difference was seen in the JC for the same genus (9.4% KB versus 2.0% MB, *t*_53_ = 5.588, *P* = 0.00001) (Fig. 5B).

Two *Alphaproteobacteria* taxa were significantly higher in the JCs of Maunalua Bay than in those of Kaneohe Bay (*Methylocystaceae* [family], 1.8% MB versus 0.5% KB, *t*_53_ = 3.639, *P* = 0.01; *Rhizobiales* [order], 0.6% MB versus 0.3% KB, *t*_53_ = 3.107, *P* = 0.04), while a third (*Leisingera* sp.) was significantly higher in the Kaneohe Bay JCs than in the Maunalua Bay JCs (0.01% MB versus 0.8% KB, *t*_53_ = 3.085, *P* = 0.04) (Fig. 5B). However, the ANGs showed no differences in these specific *Alphaproteobacteria* groups between the two populations. These changes within the JC *Alphaproteobacteria* taxa indicate that this group may also shift slightly between the populations, although none of these individual taxa account for substantial proportions of the communities. The *Leisingera* sp. differences in this study between MB and KB JCs appear to be due in large part to a few outliers within the Kaneohe Bay JCs (Fig. 5B). Previous research demonstrated that a majority of the *Rhodobacteraceae* found in the E. scolopes ANG/JC community belonged to the *Leisingera* genus (2, 3, 7, 15); however, the 16S rRNA gene V4 region does not provide enough resolution to consistently resolve *Rhodobacteraceae* genera. The lower reported values of *Leisingera* in the MB ANGs and JCs in this study than those previously published for the same samples (3) are due in part to this lack of resolution in the V4 region of the 16S rRNA gene, as well as to the use of *de novo* clustering instead of reference-based OTU clustering.

## DISCUSSION

In this study, we find that the bobtail squid ANG MB and KB bacterial communities are largely similar, with small yet significant changes between some members. The ANG and JC communities were dominated by *Alphaproteobacteria* from the *Rhodobacteraceae* family and *Verrucomicrobia* from the *Opitutae* class in both locations (Fig. 4). Alpha diversity metrics showed no differences between the populations, demonstrating that the communities are similar in terms of species richness, evenness, and phylogenetic diversity. However, beta diversity analysis revealed that samples from Kaneohe Bay exhibited tighter dispersion, clustering apart from the Maunalua Bay samples but still contained within the larger MB group, indicating a distinct but similar community composition (Fig. 2 and 3). The Kaneohe Bay population contained a significantly higher proportion of *Gammaproteobacteria* from the BD2-13 genus (a member of the *Alteromonadaceae*) (Fig. 5). Altogether, these data suggest that the community is stable between these two host populations, with the slight differences in community composition potentially reflecting local adaptation to differing environmental conditions, localized variability, or functional redundancy between the members.

The main taxonomic difference between the MB and KB ANG communities was due to an increase in relative abundance of *Alteromonadaceae* in the KB population. *Alteromonadaceae* are known to produce many secondary metabolites with antibacterial and anticancer properties (16, 17). Secondary metabolite production by symbiotic bacteria is hypothesized to contribute to cephalopod egg defense from potential fouling and infection during development (2, 6–8), although this has yet to be demonstrated *in vivo*. The potential for functional redundancy between alteromonads and other bacterial groups in the ANG could also provide further insight into how this bacterial consortium contributes to egg defense. The two host populations in MB and KB are known to contain different light organ strains of V. fischeri (12). However, colonization efficiency is not affected by the source population of squid or V. fischeri symbionts, suggesting a lack of host-symbiont coevolution (13) and functional redundancy between the strains. The higher prevalence of *Alteromonadaceae* in the KB ANG symbiosis should be explored further and may provide a novel source for natural product discovery. While *Actinobacteria* and to a lesser degree *Cyanobacteria* have been responsible for the majority of natural product drug discovery in marine ecosystems, the *Alteromonadaceae* are responsible for a high proportion of natural products derived from *Proteobacteria*, especially compared to the *Alphaproteobacteria* (16–18).

The minor differences found in the ANG community composition between these two populations may be potentially tied to morphological differences already described for these squid. MB day 0 eggs are known to be larger than KB day 0 eggs (3.5-mm diameter versus 3.0-mm diameter, respectively [11]). This size difference could indicate a larger amount of jelly coat in the MB eggs and thus a higher overall abundance of bacteria, or alternatively may be due to a larger yolk sac and/or embryo. Differences in bacterial communities caused by abundance and/or strain differences may influence the defensive potential (i.e., secondary metabolite production) of eggs from one population versus the other. Further research comparing E. scolopes ANG/JC populations from other geographic locations and comparisons between cephalopod species may answer some of these questions.

While functional redundancy may explain the variation in communities found between these two populations, differing environmental conditions might also play a role. Kaneohe Bay is a barrier reef, while Maunalua Bay is a fringing reef, and thus, these two sites are subjected to different types of water currents. Both sites contain a high level of nonindigenous and cryptogenic species (KB, 18.8%; MB, 18% of total biota), as well as invasive macroalgae (19, 20). However, the observed species distributions at sites of E. scolopes collections are distinct, with the highest number of macrofaunal taxa found in Kaneohe Bay belonging to polychaeta and gastropods, while in Maunalua Bay amphipods and red algae dominate (19, 20). These ecosystems could exert different predation and fouling pressures on bobtail squid eggs, leading to local adaptations of the symbiont populations. Additionally, distinct bacterial communities in the KB seawater and sediment could provide different source populations for the environmental transmission of the community. The developing squid are hypothesized to reacquire their ANG symbionts every generation from the environment (3), and so variation in symbiont availability between KB and MB may influence the ANG community compositions of these two populations.

In many other host-microbe associations, symbionts have been found to diverge between different populations. Gut communities frequently vary due to differences in diet between populations, as is observed in the human gut microbiome (21, 22) or in the juvenile Atlantic salmon, Salmo salar, where the gut microbiome varied between populations only in *Mycoplasmataceae* strains (23). The hindgut microbiota of termites, Reticulitermes flavipes, from different but nearby populations, showed similar abundance patterns for the core taxa but did exhibit variation hypothesized to allow the termites to distinguish nestmates from invaders (24). Furthermore, obligate nutritional endosymbionts, such as *Symbiodinium* in corals, can vary at the strain level (25).

In a symbiosis that may be functionally similar to that of the ANG, the epithelial bacterial community of Hydra oligactis has been shown to provide protection from fungal fouling (26). The H. oligactis epithelial symbiosis and the ANG symbiosis appear to share similar population dynamics. A comparison of Hydra oligactis populations from two German lakes found that the populations contained many of the same bacterial taxa and grouped together apart from the community of Hydra vulgaris from one of the same lakes (27). However, each population did contain some bacterial taxa not found in the other population (27), similar to what we observed in this study for the two populations of E. scolopes.

Despite the population differences, the overall ANG community dynamics within each host population appear to be similar. We found no dissimilarity between the overall ANG and JC community composition, and the JC bacterial community of a given female’s eggs clustered with its corresponding ANG (Fig. 2), providing additional evidence for the deposition of the ANG bacterial community into the egg JC. Expanding on the conclusions reached for the MB population (3), comparison of the ANG communities from genetically isolated host populations reinforces the hypothesis that ANG symbionts are taxonomically conserved in this and other cephalopod species. The conserved bacterial taxa between these isolated populations lead us to predict that similar ANG symbiotic communities will be found across populations of E. scolopes. Previous studies suggest that similar bacterial taxa are shared between E. scolopes and other ANG-containing cephalopods (28–31). *Alphaproteobacteria* appear to make up the majority of the taxa found in these symbioses, along with *Gammaproteobacteria* to a lesser extent (28–31). Future studies should also focus on determining whether functional conservation exists between the different bacterial strains found in various cephalopods.

The selective pressure exerted on a defensive symbiosis will largely depend on the abundance and fitness effects of specific pathogens/foulers in the host’s natural environment (32–35). If fouling only rarely impacts E. scolopes clutches, or if that fouling does not negatively impact host survival or fitness, then the selective pressure to conserve the symbiosis throughout the species should be low. Distinct environmental conditions between populations could result in different selective pressures. The largely conserved ANG symbiosis between the MB and KB bobtail squid populations may reflect the strong threat of egg fouling or infection by marine microbes. In the future, *in situ* experiments investigating fouling of eggs where the JC community has been altered (e.g., by antibiotic treatment) may lend insight into the occurrence of this threat in the host’s natural environment. Examination of bobtail squid populations from other islands in the Hawaiian archipelago will also enhance our understanding of the stability of the ANG community across the species. A previous genome study of roseobacters isolated from the ANG of MB E. scolopes suggested that there are differences between closely related strains (15). Future work on specific KB ANG isolates along with metagenomic and transcriptomic studies may lead to a better understanding of these differences. The overall stability of the community between host populations supports a critical functional role for this symbiosis, while the few variable taxa open up potential avenues for understanding how local host-microbe populations adapt to different conditions and for isolating additional drug discovery candidates.

## MATERIALS AND METHODS

Ten sexually mature female squid (ranging in mantle length from 19 mm to 30 mm) were collected from Kaneohe Bay (KB, 21°16′51.42″N, 57°43′33.07″W) using dip nets and were immediately transferred to Kewalo Marine Laboratory, Oahu, HI. Squid either were sacrificed within 2 days or were shipped to Connecticut and maintained in our squid facility for up to 4 months. Lab-maintained females were regularly mated and kept in individual tanks to allow clutches to be matched to the individual mothers. Bobtail squid were anesthetized in 2% ethanol in artificial seawater prior to sacrifice. Egg clutches were collected and dissected within 12 h of deposition. All samples were surface sterilized in 99% ethanol and filter-sterilized squid Ringer’s solution (FSSR [2]) to remove transient bacterial contaminants.

DNA extraction from ANGs (*n* = 10) and egg JCs (*n* = 20) was completed as previously described (3). Briefly, ANGs were homogenized in FSSR, followed by differential centrifugation to separate the bacterial cells from host tissue. DNA extraction of the bacterial component was completed using the DNeasy Blood and Tissue kit (Qiagen, Valencia, CA) with bead beating (Mini-Beadbeater-16; BioSpec Products, Bartlesville, OK). Ten JCs were separated from their outer egg capsules and yolk sacs and pooled in a bead-beating tube. The JCs were flash-frozen to −80°C for a minimum of 30 min, and DNA was extracted using the MasterPure DNA purification kit (Epicentre Biotechnologies, Madison, WI) with bead beating and an increased concentration of proteinase K (0.833 µg/ml).

Extracted DNA was amplified using bar-coded primers developed for the V4 region of the 16S rRNA gene by Caporaso et al. (36) and sequenced on an Illumina MiSeq sequencer (Illumina, San Diego, CA) according to established protocols (24, 37). Samples were processed either in the Nyholm lab or at the University of Connecticut Microbial Analysis, Resources and Services facility (MARS). An average of 50,052 ± 12,197 reads/sample was obtained for KB ANG samples (*n* = 10, minimum 23,976 reads/sample). An average of 66,550 ± 32,128 reads/sample was obtained for KB JCs (*n* = 20, minimum 17,654 reads/sample). MB samples were previously published and reanalyzed for this study and contained an average of 82,077 ± 31,037 reads/sample for the JC and 74,739 ± 31,370 reads/sample for the ANG (3). Both negative-extraction (no-sample) and PCR (no-template) controls were processed and sequenced simultaneously with all samples. Fewer than 1,000 sequences/control were obtained in all cases, and the majority of sequences in these controls belonged to a single *Escherichia* OTU. Most other OTUs present in the controls were not present in the ANG samples. Three *Rhodobacteraceae* OTUs also associated with the community were obtained in the controls as well but accounted for less than 1% of sequences for the control samples. In addition, the presence of *Rhodobacteraceae* in the ANG has been previously established through the use of fluorescence *in situ* hybridization (2) and culturing techniques (15). No *Verrucomicrobia* OTUs were found in any of the control samples.

Sequencing data were analyzed using QIIME (38). *De novo* methods were used to assign operational taxonomic units (OTUs) at the 97% identity level (24). Samples were rarefied to 10,000 sequences. Alpha diversity was analyzed in QIIME, and the log_2_ Shannon index was converted to a natural log Shannon index. Alpha diversity plots were created, and differences in alpha diversity were tested using two-way ANOVA with *post hoc* Tukey tests in Prism. Beta diversity was analyzed using the Bray-Curtis metric, with community composition similarity tested by ANOSIM and PERMANOVA in QIIME and NMDS plots created in R using the Vegan package (39). Differences in relative abundance between various taxa were analyzed by unpaired *t* test and corrected for multiple comparisons using the Holm-Sidek method in Prism. KB sequences were compared to MB sequences previously published and available under project identifier (ID) PRJEB14655, accession numbers ERS1498392 to ERS1498398, ERS1496666 to ERS1496676, and ERS1496678 to ERS1496722 (3). MB sequences were reanalyzed for this study for consistency.

### Accession number(s).

KB sequences were deposited in the European Nucleotide Archive (ENA) and are available under project ID PRJEB23264.

## References

[1] Jones BW, Nishiguchi MK 2004 Counterillumination in the Hawaiian bobtail squid, *Euprymna scolopes* Berry (*Mollusca*: *Cephalopoda*). Mar Biol 144:1151–1155. doi:10.1007/s00227-003-1285-3.

[2] Collins AJ, LaBarre BA, Wong Won BS, Shah MV, Heng S, Choudhury MH, Haydar SA, Santiago J, Nyholm SV 2012 Diversity and partitioning of bacterial populations within the accessory nidamental gland of the squid *Euprymna* *scolopes*. Appl Environ Microbiol 78:4200–4208. doi:10.1128/AEM.07437-11. 22504817 PMC3370523

[3] Kerwin AH, Nyholm SV 2017 Symbiotic bacteria associated with a bobtail squid reproductive system are detectable in the environment, and stable in the host and developing eggs. Environ Microbiol 19:1463–1475. doi:10.1111/1462-2920.13665. 28063183

[4] Buchner P 1965 Endosymbiosis of animals with plant microorganisms, p 543–571. Interscience Publishers, New York, NY.

[5] Döring W 1908 Über bau und entwicklung des weiblichen geschlechts-apparates bei myopsiden cephalopodan. Z Wiss Zool 91:112–189.

[6] Biggs J, Epel D 1991 Egg capsule sheath of *Loligo* *opalescens* Berry: structure and association with bacteria. J Exp Zool 259:263–267. doi:10.1002/jez.1402590217.

[7] Gromek SM, Suria AM, Fullmer MS, Garcia JL, Gogarten JP, Nyholm SV, Balunas MJ 2016 *Leisingera* sp. JC1, a bacterial isolate from Hawaiian bobtail squid eggs, produces indigoidine and differentially inhibits vibrios. Front Microbiol 7:1342. doi:10.3389/fmicb.2016.01342. 27660622 PMC5014874

[8] Barbieri E, Barry K, Child A, Wainwright N 1997 Antimicrobial activity in the microbial community of the accessory nidamental gland and egg cases of *Loligo* *pealei* (*Cephalopoda*: *Loliginidae*). Biol Bull 193:275–276. doi:10.1086/BBLv193n2p275. 28575614

[9] Alison Kay E, Palumbi SR 1987 Endemism and evolution in Hawaiian marine invertebrates. Trends Ecol Evol 2:183–186. doi:10.1016/0169-5347(87)90017-6. 21227847

[10] Lee KH, Ruby EG 1994 Effect of the squid host on the abundance and distribution of symbiotic *Vibrio fischeri* in nature. Appl Environ Microbiol 60:1565–1571. 16349257 10.1128/aem.60.5.1565-1571.1994 PMC201518

[11] Kimbell JR, McFall-Ngai MJ, Roderick GK 2002 Two genetically distinct populations of bobtail squid, *Euprymna scolopes*, exist on the island of O’ahu. Pac Sci 56:347–355. doi:10.1353/psc.2002.0024.

[12] Wollenberg MS, Ruby EG 2009 Population structure of *Vibrio fischeri* within the light organs of *Euprymna scolopes* squid from two Oahu (Hawaii) populations. Appl Environ Microbiol 75:193–202. doi:10.1128/AEM.01792-08. 18997024 PMC2612210

[13] Bongrand C, Koch EJ, Moriano-Gutierrez S, Cordero OX, McFall-Ngai M, Polz MF, Ruby EG 2016 A genomic comparison of 13 symbiotic *Vibrio fischeri* isolates from the perspective of their host source and colonization behavior. ISME J 10:2907–2917. doi:10.1038/ismej.2016.69. 27128997 PMC5148191

[14] Wollenberg MS, Ruby EG 2012 Phylogeny and fitness of *Vibrio fischeri* from the light organs of *Euprymna scolopes* in two Oahu, Hawaii populations. ISME J 6:352–362. doi:10.1038/ismej.2011.92. 21776028 PMC3260510

[15] Collins AJ, Fullmer MS, Gogarten JP, Nyholm SV 2015 Comparative genomics of Roseobacter clade bacteria isolated from the accessory nidamental gland of *Euprymna scolopes*. Front Microbiol 6:123. doi:10.3389/fmicb.2015.00123. 25755651 PMC4337385

[16] Jensen PR, Fenical W 1994 Strategies for the discovery of secondary metabolites from marine bacteria: ecological perspectives. Annu Rev Microbiol 48:559–584. doi:10.1146/annurev.mi.48.100194.003015. 7826019

[17] Desriac F, Jégou C, Balnois E, Brillet B, Le Chevalier P, Fleury Y 2013 Antimicrobial peptides from marine *Proteobacteria*. Mar Drugs 11:3632–3660. doi:10.3390/md11103632. 24084784 PMC3826127

[18] Williams PG 2009 Panning for chemical gold: marine bacteria as a source of new therapeutics. Trends Biotechnol 27:45–52. doi:10.1016/j.tibtech.2008.10.005. 19022511

[19] Coles SL, DeFelice RC, Eldredge LG 2002 Nonindigenous marine species in Kane’ohe Bay, O’ahu, Hawai’i. Bishop Museum technical report no. 24. Bishop Museum Press, Honolulu, HI.

[20] Coles S, DeFelice R, Eldredge L 2002 Nonindigenous marine species at Waikiki and Hawai’i Kai, O’ahu, Hawai’i. Bishop Museum technical report no. 25. Bishop Museum Press, Honolulu, HI.

[21] Yatsunenko T, Rey FE, Manary MJ, Trehan I, Dominguez-Bello MG, Contreras M, Magris M, Hidalgo G, Baldassano RN, Anokhin AP, Heath AC, Warner B, Reeder J, Kuczynski J, Caporaso JG, Lozupone CA, Lauber C, Clemente JC, Knights D, Knight R, Gordon JI 2012 Human gut microbiome viewed across age and geography. Nature 486:222–227. doi:10.1038/nature11053. 22699611 PMC3376388

[22] Lozupone CA, Stombaugh JI, Gordon JI, Jansson JK, Knight R 2012 Diversity, stability and resilience of the human gut microbiota. Nature 489:220–230. doi:10.1038/nature11550. 22972295 PMC3577372

[23] Llewellyn MS, McGinnity P, Dionne M, Letourneau J, Thonier F, Carvalho GR, Creer S, Derome N 2016 The biogeography of the Atlantic salmon (*Salmo salar*) gut microbiome. ISME J 10:1280–1284. doi:10.1038/ismej.2015.189. 26517698 PMC5029221

[24] Benjamino J, Graf J 2016 Characterization of the core and caste-specific microbiota in the termite, *Reticulitermes flavipes*. Front Microbiol 7:171. doi:10.3389/fmicb.2016.00171. 26925043 PMC4756164

[25] Frade PR, De Jongh F, Vermeulen F, Van Bleijswijk J, Bak RPM 2008 Variation in symbiont distribution between closely related coral species over large depth ranges. Mol Ecol 17:691–703. doi:10.1111/j.1365-294X.2007.03612.x. 18179427

[26] Fraune S, Anton-Erxleben F, Augustin R, Franzenburg S, Knop M, Schröder K, Willoweit-Ohl D, Bosch TC 2015 Bacteria-bacteria interactions within the microbiota of the ancestral metazoan *Hydra* contribute to fungal resistance. ISME J 9:1543–1556. doi:10.1038/ismej.2014.239. 25514534 PMC4478695

[27] Fraune S, Bosch TCG 2007 Long-term maintenance of species-specific bacterial microbiota in the basal metazoan *Hydra*. Proc Natl Acad Sci U S A 104:13146–13151. doi:10.1073/pnas.0703375104. 17664430 PMC1934924

[28] Barbieri E, Paster BJ, Hughes D, Zurek L, Moser DP, Teske A, Sogin ML 2001 Phylogenetic characterization of epibiotic bacteria in the accessory nidamental gland and egg capsules of the squid *Loligo* *pealei* (*Cephalopoda*: *Loliginidae*). Environ Microbiol 3:151–167. doi:10.1046/j.1462-2920.2001.00172.x. 11321532

[29] Grigioni S, Boucher-Rodoni R, Demarta A, Tonolla M, Peduzzi R 2000 Phylogenetic characterisation of bacterial symbionts in the accessory nidamental glands of the sepioid *Sepia officinalis* (*Cephalopoda*: *Decapoda*). Mar Biol 136:217–222. doi:10.1007/s002270050679.

[30] Pichon D, Gaia V, Norman MD, Boucher-Rodoni R 2005 Phylogenetic diversity of epibiotic bacteria in the accessory nidamental glands of squids (*Cephalopoda*: *Loliginidae* and *Idiosepiidae*). Mar Biol 147:1323–1332. doi:10.1007/s00227-005-0014-5.

[31] Lum-Kong A, Hastings TS 1992 The accessory nidamental glands of *Loligo* *forbesi* (*Cephalopoda*: *Loliginidae*): characterization of symbiotic bacteria and preliminary experiments to investigate factors controlling sexual maturation. J Zool 228:395–403. doi:10.1111/j.1469-7998.1992.tb04443.x.

[32] Smith AH, Łukasik P, O’Connor MP, Lee A, Mayo G, Drott MT, Doll S, Tuttle R, Disciullo RA, Messina A, Oliver KM, Russell JA 2015 Patterns, causes and consequences of defensive microbiome dynamics across multiple scales. Mol Ecol 24:1135–1149. doi:10.1111/mec.13095. 25683348

[33] Oliver KM, Smith AH, Russell JA 2014 Defensive symbiosis in the real world—advancing ecological studies of heritable, protective bacteria in aphids and beyond. Funct Ecol 28:341–355. doi:10.1111/1365-2435.12133.

[34] Parker BJ, Barribeau SM, Laughton AM, de Roode JC, Gerardo NM 2011 Non-immunological defense in an evolutionary framework. Trends Ecol Evol 26:242–248. doi:10.1016/j.tree.2011.02.005. 21435735

[35] Jaenike J, Unckless R, Cockburn SN, Boelio LM, Perlman SJ 2010 Adaptation via symbiosis: recent spread of a *Drosophila* defensive symbiont. Science 329:212–215. doi:10.1126/science.1188235. 20616278

[36] Caporaso JG, Lauber CL, Walters WA, Berg-Lyons D, Huntley J, Fierer N, Owens SM, Betley J, Fraser L, Bauer M, Gormley N, Gilbert JA, Smith G, Knight R 2012 Ultra-high-throughput microbial community analysis on the Illumina HiSeq and MiSeq platforms. ISME J 6:1621–1624. doi:10.1038/ismej.2012.8. 22402401 PMC3400413

[37] Nelson MC, Morrison HG, Benjamino J, Grim SL, Graf J 2014 Analysis, optimization and verification of Illumina-generated 16S rRNA gene amplicon surveys. PLoS One 9:e94249. doi:10.1371/journal.pone.0094249. 24722003 PMC3983156

[38] Caporaso JG, Kuczynski J, Stombaugh J, Bittinger K, Bushman FD, Costello EK, Fierer N, Peña AG, Goodrich JK, Gordon JI, Huttley GA, Kelley ST, Knights D, Koenig JE, Ley RE, Lozupone CA, McDonald D, Muegge BD, Pirrung M, Reeder J, Sevinsky JR, Turnbaugh PJ, Walters WA, Widmann J, Yatsunenko T, Zaneveld J, Knight R 2010 QIIME allows analysis of high-throughput community sequencing data. Nat Methods 7:335–336. doi:10.1038/nmeth.f.303. 20383131 PMC3156573

[39] Oksanen J, Blanchet F, Friendly M, Kindt R, Legendre P, McGlinn D, Minchin P, O’Hara B, Simpson G, Solymos P, Stevens M, Szoecs E, Wagner H 2017 Vegan: community ecology package. R package version 2.4-4.

