# Supplementary figures and images for: Reproductive System Symbiotic Bacteria Are Conserved between Two Distinct Populations of Euprymna scolopes from Oahu, Hawaii
Source: mSphere. 2018 Mar 28;3(2):e00531-17. doi: 10.1128/mSphere.00531-17 (PMC5874440; doi:10.1128/mSphere.00531-17)

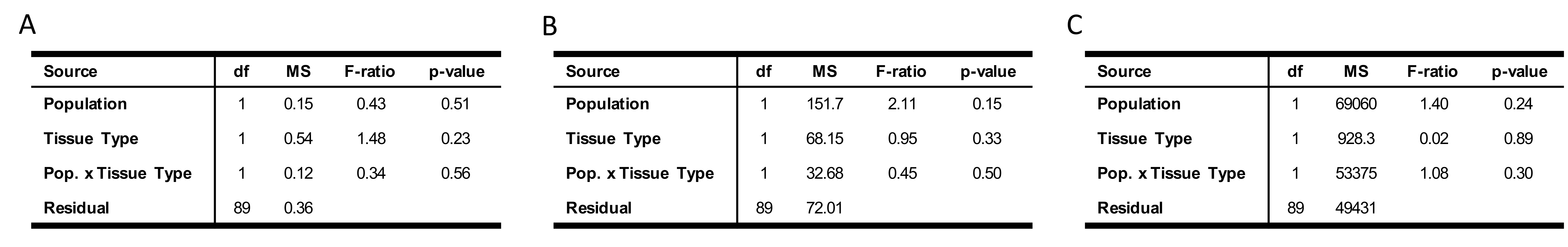

Supplement: TABLE S1 [file sph002182499st1.tif]
